# Relationship between Vascular Resistance and Sympathetic Nerve Fiber Density in Arterial Vessels in Children With Sleep Disordered Breathing

**DOI:** 10.1161/JAHA.117.006137

**Published:** 2017-07-17

**Authors:** Anna Kontos, Kurt Lushington, James Martin, Quenten Schwarz, Ryan Green, David Wabnitz, Xiangjun Xu, Elke M. Sokoya, Scott Willoughby, Mathias Baumert, Antonio Ferrante, Melissa La Forgia, Declan Kennedy

**Affiliations:** ^1^ Robinson's Research Institute School of Medicine Discipline of Paediatrics and Reproductive Health University of Adelaide Adelaide Australia; ^2^ School of Electrical and Electronic Engineering University of Adelaide Adelaide Australia; ^3^ Department of Immunology SA Pathology Schools of Medicine and Biological Science University of Adelaide Adelaide Australia; ^4^ School of Psychology Social Work and Social Policy University of South Australia Adelaide Australia; ^5^ Neurovascular Research Laboratory Centre for Cancer Biology University of South Australia Adelaide Australia; ^6^ Department of Information Technology, Engineering and the Environment University of South Australia Adelaide Australia; ^7^ Department of Respiratory and Sleep Medicine Women's and Children's Hospital Adelaide Australia; ^8^ Department of Otolaryngology–Head and Neck Surgery Women's and Children's Hospital Adelaide Australia; ^9^ Department of Medical Imaging Women's and Children's Hospital Adelaide Australia; ^10^ Department of Human Physiology Flinders University Adelaide South Australia Australia; ^11^ Centre for Heart Rhythm Disorders South Australian Health and Medical Research Institute University of Adelaide and Royal Adelaide Hospital Adelaide Australia

**Keywords:** arterial blood flow, arterial stiffness, autonomic function, flow‐mediated dilatation, pediatrics, shear stress, sleep disordered breathing, sympathetic, sympathetic nerve fibre density, vasculature, Vascular Biology, Vascular Disease, Atherosclerosis, Hypertension, Pediatrics

## Abstract

**Background:**

Sleep disordered breathing in children is associated with increased blood flow velocity and sympathetic overactivity. Sympathetic overactivity results in peripheral vasoconstriction and reduced systemic vascular compliance, which increases blood flow velocity during systole. Augmented blood flow velocity is recognized to promote vascular remodeling. Importantly, increased vascular sympathetic nerve fiber density and innervation in early life plays a key role in the development of early‐onset hypertension in animal models. Examination of sympathetic nerve fiber density of the tonsillar arteries in children undergoing adenotonsillectomy for Sleep disordered breathing will address this question in humans.

**Methods and Results:**

Thirteen children scheduled for adenotonsillectomy to treat sleep disordered breathing underwent pupillometry, polysomnography, flow‐mediated dilation, resting brachial artery blood flow velocity (velocity time integral), and platelet aggregation. The dorsal lingual artery (tonsil) was stained and immunofluorescence techniques used to determine sympathetic nerve fiber density. Sympathetic nerve fiber density was correlated with increased resting velocity time integral (*r*=0.63; *P*<0.05) and a lower Neuronal Pupillary Index (*r*=−0.71, *P*<0.01), as well as a slower mean pupillary constriction velocity (mean, *r*=−0.64; *P*<0.05). A faster resting velocity time integral was associated with a slower peak pupillary constriction velocity (*r*=−0.77; *P*<0.01) and higher platelet aggregation to collagen antigen (*r*=0.64; *P*<0.05). Slower mean and peak pupillary constriction velocity were associated with higher platelet aggregation scores (*P*<0.05; *P*<0.01, respectively).

**Conclusions:**

These results indicate that sympathetic activity is associated with change in both the function and structure of systemic vasculature in children with sleep disordered breathing.

**Clinical Perspective**

**What Is New?**

- Children with sleep disordered breathing and increased blood flow velocity have increased sympathetic activity, increased vascular sympathetic nerve fiber density, and increased markers of endothelial damage.

**What Are the Clinical Implications?**

- These functional and structural changes may be significant precursor conditions of adult hypertension and cardiovascular disease.

## Introduction

In adults, sympathetic tone is an important factor in vascular health, with increased tone adversely affecting vascular function and leading over time to hypertension and vascular remodeling.^1^ Increased sympathetic tone is also a feature of several childhood disorders,^2^, ^3^ including sleep disordered breathing (SDB).^4^, ^5^, ^6^ SDB affects an estimated 10% of children, but is often underdiagnosed and is associated with hypertension, which is thought to reflect the effect of a persistent increase in sympathetic tone.^7^ In adults with SDB, there is evidence that the effects of sympathetic overactivity on blood vessels is systemic.^8^ Our group and others have postulated that some children with SDB also develop systemic changes in vascular compliance, which arise in direct response to increased sympathetic activity.^9^, ^10^

In SDB, partial or total collapse of the upper airway leads to increased respiratory effort, intrathoracic pressure swings, intermittent hypoxia, sleep fragmentation, and increased arousals.^11^ These factors lead to sympathetic overactivation with the release of noradrenaline from vascular sympathetic nerve fiber terminals and a consequent increase in heart rate, inotropy, and peripheral vasoconstriction.^12^ In turn, the reduced vascular compliance and concomitant increase in total peripheral resistance^13^ leads to an increase in blood flow velocity during systole and thereby also increases luminal shear stress.^14^, ^15^, ^16^, ^17^ A persistent increase in shear stress damages the vascular endothelial cells surrounding the lumen and increases platelet aggregation.^18^ Sympathetic overactivation is also known to promote vascular cell proliferation (smooth muscle cells) and increase the production of extracellular matrix proteins, such as elastin and collagen.^19^, ^20^ Importantly, in animal models, early exposure to sympathetic hyperactivity is associated with development of early‐onset hypertension^21^ and increased recruitment of peripheral vascular sympathetic neuronal fibers (density).^21^, ^22^, ^23^, ^24^

We have previously reported altered cardiovascular function in children with SDB, including: an accelerated heart rate response to cortical arousals^25^ and a delayed hyperemic dilatation response and increased blood flow velocity, both at rest and during hyperemia, in the brachial artery suggestive of reduced vascular compliance.^9^ Others have also reported abnormal vascular function in all severity levels of the disorder compared with nonsnoring children, including reduced dilation capacity, reduced vascular compliance, and increased blood flow velocity.^26^, ^27^, ^28^, ^29^ But, to date, the relationship between sympathetic activity and its effects on vascular function (blood flow velocity, arterial dilation capacity, and endothelium integrity) and vascular structure in children with SDB are unknown. This question may be answered by an examination of tonsillar blood vessels from children undergoing adenotonsillectomy for SDB, who have also previously undergone vascular functional testing (brachial artery blood flow velocity, flow‐mediated dilation [FMD] and platelet aggregation). We hypothesize that the functional changes associated with sympathetic overactivation in children with SDB will be associated with changes in vascular structural—that is, increased tonsillar artery sympathetic nerve fiber density (SNFD). Adenotonsillectomy offers a unique opportunity to address this assumption by histologically evaluating tonsillar blood vessels for evidence of changes in sympathetic innervation and to match the histological findings with accepted functional markers of altered autonomic tone.^30^, ^31^, ^32^

In summary, the aim of this study was to utilize vascular sympathetic nerve density as a structural measure of sympathetic innervation and to correlate this with a functional measure of autonomic tone (pupillometry) and the functional vascular response observed in FMD and platelet aggregation in children who were scheduled for adenotonsillectomy for the treatment of SDB.

## Methods

Children with a history of parental report of snoring more than 3 nights per week, and who were referred by their primary physician for the evaluation of potential SDB by experienced otolaryngologists at the Women's and Children's Hospital Adelaide, Australia, and who were subsequently scheduled for adenotonsillectomy were recruited as participants (n=15). Children with a history of significant asthma, previous adenoidectomy, evidence of recent tonsillar infection (parental report), craniofacial abnormalities, medications that affect sleep or respiration, and English as a second language were excluded from the study. The study was approved by the Women's and Children's Hospital and University of Adelaide's Human Research Ethics Committees and has been performed in accord with the ethical standards laid down in the 1964 Declaration of Helsinki and its later amendments. All parents of children in this study provided written consent and children also provided written assent.

### Anthropometrics

Children were weighed wearing minimal clothing using an electronic scale with a resolution of 0.1 kg, and height was measured using a wall‐mounted stadiometer. Body mass index and percentile was calculated using the Baylor College of Medicine website tool and adjusted for age and sex (http://www.bcm.edu/cnrc/bodycomp/bmiz2.html).

### Polysomnography

Overnight PSG was conducted using the Compumedics E‐Series Sleep System (Melbourne, Australia).^9^ The following standard measures were collected: EEG (C3‐A2, C4‐A1, and F3‐A2 F4‐A1, O1‐A2, and O2‐A1), left and right EOG, submental, diaphragmatic, and leg EMG, heart rate by ECG, oronasal airflow by thermistor and nasal pressure, respiratory movements of the chest and abdominal wall using uncalibrated respiratory inductive plethysmography, and arterial oxygen saturation by pulse oximetry (Nellcor N‐595; 2‐second averaging time). Children were continuously monitored by infrared camera by a pediatric sleep technician who also documented observations of sleep behavior, which included the presence or absence of snoring. PSG records were scored by an experienced sleep technician blind to subject status and according to established sleep stage^33^ and ventilatory criteria.^34^

### Pupillometry

The pupillary light reflex was used to evaluate autonomic tone.^32^, ^35^, ^36^, ^37^ Pupillary response was measured using the handheld NeurOptics device (NeuroOptics, Irvine, CA). The device consists of an infrared illumination source and a digital camera to capture pupillary images and an onboard processor with summary data output to a liquid crystal display. The device delivers a light pulse to stimulate the eye and then analyzes subsequent changes in pupil diameter from a succession of digital images.

The pupillary light reflex was measured before sleep onset at a time when the sympathetic nervous system is relatively quiescent and parasympathetic vagal control is dominant.^32^, ^38^ Specifically, on completion of the setup for the overnight PSG, the room light was dimmed to a standard lux (8–9 lux), and children were instructed to lie supine in their beds for 5 minutes. Children were then instructed to focus on a fixed point on the ceiling and their pupillary response to a 180‐μW, 33‐ms light pulse was sampled at 30 frames per second for 3 seconds in each eye consecutively.

The following pupillary parameters were obtained from the NeurOptics device: (1) baseline pupillary diameter (maximal diameter); (2) constricted diameter at peak of the light reflex (minimal diameter); (3) percentage change in pupil diameter from baseline; (4) constriction latency (time difference between initiation of retinal light stimulation and onset of pupillary constriction); (5) average constriction velocity (amount of constriction/duration of constriction mm/s); and (6) maximal constriction velocity (peak value of velocity during constriction) and postconstriction velocity (dilation velocity after peak constriction). Using proprietal software, the above variables were compared to a normative pupillary light reflex data set to derive a Neurological Pupil Index graded on a 1‐ to 5‐point scale with a score <3 being indicative of an abnormal pupillary response.^39^ Normative outlines for pupillometry are found in Boev et al.^40^

### Flow‐Mediated Dilation

FMD was measured upon waking following overnight PSG and while still fasting.^9^, ^41^ The brachial artery diameter was determined from a 30‐second resting scan measured perpendicular to the brachial artery and 10 cm proximal to the elbow. The transducer was then set on B mode and positioned at a 60‐degree angle to the vessel, and subsequent blood flow dynamics were collected (resting peak systolic velocity [PSV], resting velocity time integral [VTi] and heart rate). A minimum of 6 image sets were taken, and averaged values were used to determine resting blood flow dynamics. The lower forearm was then occluded by inflating a sphygmomanometer cuff to 200 mm Hg for 4 minutes. A subsequent 3‐minute scan was taken, beginning 15 seconds before cuff removal and saved as a dicom file.

**Figure 1:**
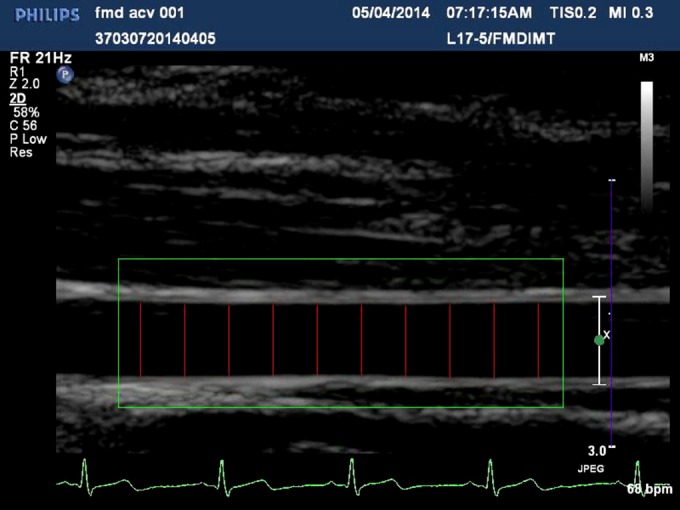
Sonogram of the brachial artery, 10 cm proximal from the elbow, green box determines the region of interest. Ten equidistant red lines were used to determine the frame by frame diameter difference over 180 seconds (15 seconds precuff release until 165 seconds).

**Figure 2:**
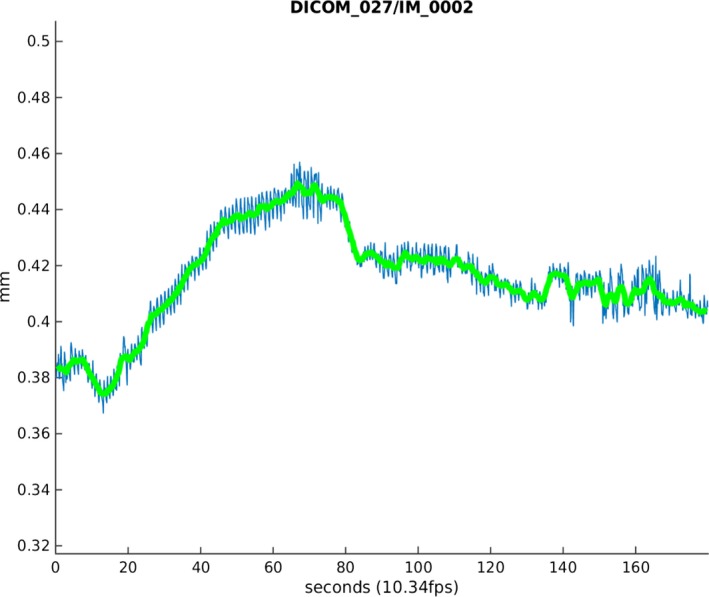
Example of the mean dilatation response from a child with sleep disordered breathing. The computer program generates a graph showing the raw data in blue and the averaged data in green.

A customized Matlab (2016B; The MathWorks, Inc, Natick, MA) program was used to determine regions of interest (ROIs) from the whole brachial vessel. The ROI window was manually manipulated for each individual vessel so that it remained within the ROI window for the duration of the scan (3 minutes—2004 frames). Pixel‐to‐mm ratios were determined by the hardcoded dicom millimeter key. Ten perpendicular, evenly spaced interrogation points along the interior of the vessel were used for averaging the dilation response per frame. A pixel intensity threshold (40 from 8‐bit intensity values) was chosen by visual analysis of intensity gradients at the borders of the vessels (note: images have high spatial intensity homogeneity and consistent intensity between individuals). At each of the 10 interrogation points, the dilation of the vessel was measured by extending a line along the positive and negative y‐axis until the threshold value was reached, indicating the end of the vessel interior (Figure 1). Vessel dilation measurements in mm were calculated using the dicom and mm:pixel ratio. The interior of the vessel was then measured again from the same location in the following frame and compared to the previous measurement. To exclude values attributed to movement artifacts, measurements that exceeded the previous corresponding measurement by 10% were included as “not a number” in analysis until a subsequent measurement fell within the threshold of the last valid measurement, because they were considered outliers. A 2‐dimensional matrix consisting of 10 rows, 1 per interrogation point, and columns determined by the number of frames were used to hold all the vessel measurements generated from 1 dicom image set. A mean dilation for each frame (10 vessel measurements/10) was then determined. These values were then plotted against a time axis and used to determine the maximal diameter (FMD_Max_), the diameter at 60 seconds postcuff deflation (FMD_60S_), and time to maximal dilation (FMD_Time‐to‐Max_; Figure 2).

### Platelet Function

Blood flow is an important mediator influencing the platelet aggregation process. It has been previously proposed that different shear conditions provide distinct patterns of aggregation. Whole‐blood impedance aggregometry is based on the principle that activated platelets stick by their surface receptors to artificial surfaces of 2 electrodes within the whole‐blood sample positioned at a determined distance between them.^42^ Hence, platelet aggregation was evaluated using a Multiplate analyzer (Dynabyte, Munich, Germany) within 45 minutes postvenipuncture as previously described.^43^ The device is considered a valid, rapid, and complete measure of platelet function. Platelet aggregation was simultaneously measured using independent sensor units, for 4 different antagonists. Rate of aggregations was measured by detecting the increase in electrical impedance generated by the aggregation of the platelets.^42^ Platelet aggregation normative values can be found in Halimeh et al.^43^

Fifteen microliters of CaCl/NaCl_2_ (37°C) solution was used as a diluent for low‐dose ADP (1:4 dilution), high‐dose ADP, thrombin receptor activating peptide, and collagen aggregation, whereas saline solution was used as a diluent for aspirin dialuminate tests. All agonists were purchased from Dynabyte. One hundred fifty microliters of whole blood were then added to the impedance channels containing diluent and incubation (2 minutes, 37°C) before the addition of 20 μL of respective platelet agonist to achieve the following final concentrations: 1.6 μmol/L of low‐dose ADP, 6.5 μmol/L of high‐dose ADP, 32 μmol/L of thrombin receptor activating peptide, and 3.2 μg of collagen aggregation and a 0.5‐mmol/L aspirin dialuminate test. Measuring time was 6 minutes for all reactions, with aggregation units (Ohms) plotted against time (minutes).

### Vascular Histology

During adenotonsillectomy, tonsils were resected using a cold steel procedure and the inferior lobes of both tonsils sutured to aid orientation. Dorsal lingual arteries were microdissected from tonsils within 30 minutes of removal and immersed in modified Zambonis fixative for 24 hours (0.2% saturated picric acid in 2% paraformaldehyde in 0.1 olL‐1 of phosphate buffer). Vessels where then permeabilized by 3×10 minute washes with DMSO followed by 3×10 minute washes with PBS and then stored in PBS with 0.01% sodium azide at 4°C.^44^

### Immunohistochemisty

**Figure 3:**
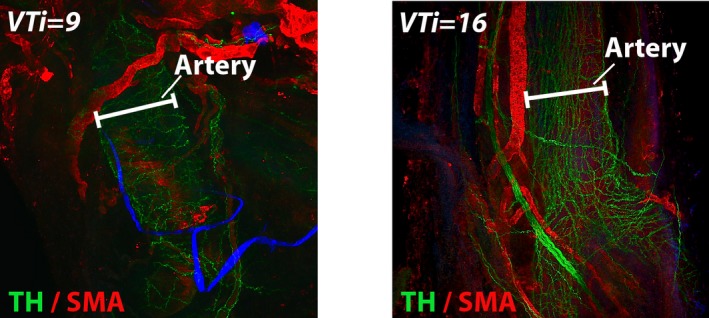
Representative images showing 2 dorsal lingual arteries from 2 children with sleep disordered breathing (left panel VTi=9 and SNFD=1803 and right panel VTi=16 and SNFD=2120). Red immunofluorescence indicates smooth muscle actin (SMA), and green immunofluorescence represents tyrosine hydroxylase (TH) an enzyme found in sympathetic nerve fibers. SNFD indicates sympathetic nerve fiber density; VTi, velocity time integral.

**Figure 4:**
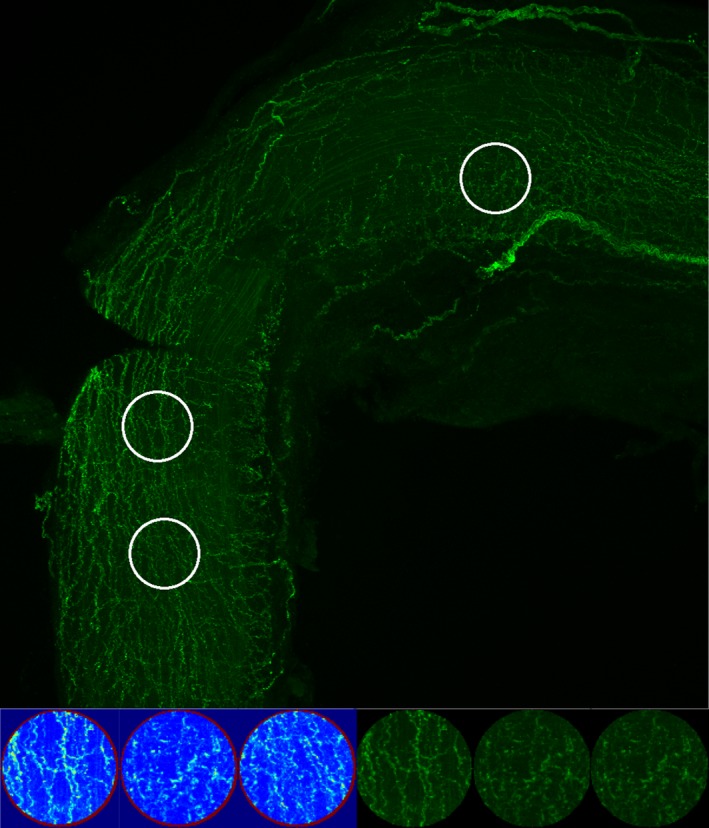
Immunofluorescence for tyrosine hydroxylase on the dorsal lingual artery; white circles indicate the 3 regions of interest selected by the analyst for green pixel/region of interest, sympathetic nerve fiber density score.

**Figure 5:**
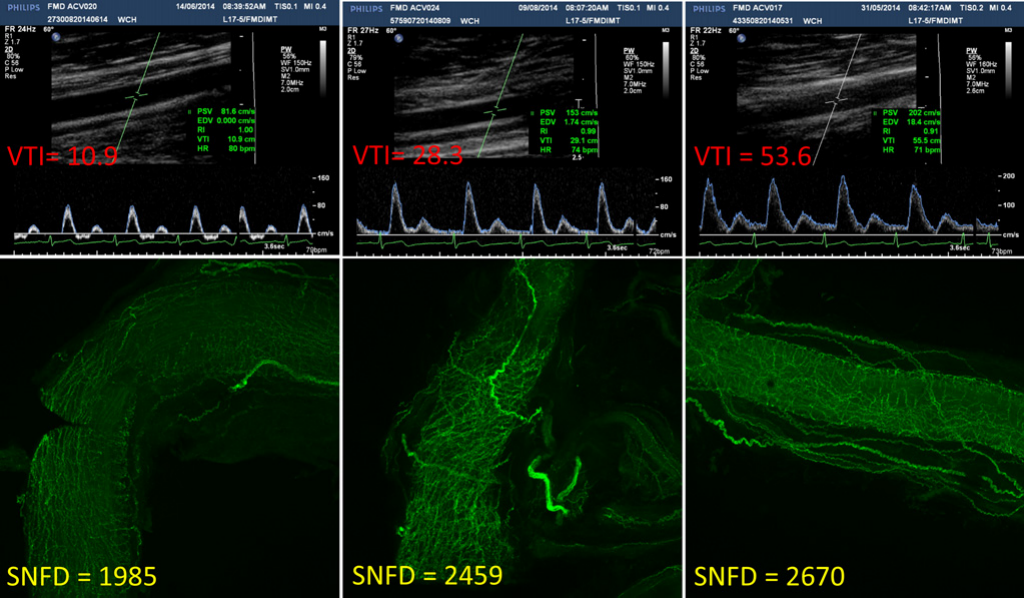
Blood flow velocity Doppler image (top panel), and the corresponding dorsal lingual arteries sympathetic nerve fiber images (bottom panel) for 3 representative participants. SNFD indicates Sympathetic Nerve Fiber Density; VTI, velocity time integral.

Vessels were blocked in PBS with 10% inactivated horse serum (Sigma‐Aldrich, St. Louis, MO) for 60 minutes at room temperature. Following removal of the blocking solution, vessels were then placed into a solution of primary antibodies diluted in PBS with 0.1% Triton‐X and 5% horse serum for 60 minutes at room temperature. Antibodies and dilutions included: anti‐mouse alpha smooth muscle actin conjugated to Alexa Fluor‐555 (1:500; Abcam, Cambridge, UK) and antirabbit tyrosine hydroxylase (1:200; Millipore, Billerica, MA). Samples were then washed 3 times with PBS before the addition of donkey antirabbit Alexa Fluor‐488–conjugated secondary antibodies (1:200; Thermo Fisher, Waltham, MA) for 60 minutes at room temperature. Whole vessels were subsequently mounted in NT_Prolong Gold antifade reagent containing DAPI (Thermo Fisher) on glass slides. All vessels were imaged using the same laser settings on a Zeiss LSM 700 Confocal microscope (Carl Zeiss, Jena, Germany) at ×5 magnification with Z‐stacks set to a depth of 2.25 to 2.85 μm. Images were captured using Zen Black software (Zeiss) and converted to PNG files (Figure 3) for image data processing using a custom algorithm (python script; Numpy for matrix analysis, Scipy for image support). Images were internally represented as 3‐dimensional matrices (x, y, rgb). Red and blue channels were discarded before immunofluorescence in the green channel was determined at 3 points along the vessel using circular interrogation regions (Figure 4) to avoid issues with rotational impartiality. Interrogation regions were chosen by an individual blinded to the results of the functional testing. They were instructed to select regions for interrogation along the vessels that were free of large nerve fiber bundles and where there was consistent SNFD in clearly imaged portions of the vessel (Figures 4 and 5). Total green values were calculated using a simple sum function of the elements in the circular region of the matrix, and the average ratio of green pixels per ROI of 3 circles was used as a measure of SNFD.

### Statistics Analysis

Data were analyzed using SPSS (IBM SPSS Statistics for Windows, Version 21.0; IBM Corp, Armonk, NY) and GraphPad Prism software (version 5.00; GraphPad Software Inc, San Diego, CA). All data are represented as mean (SD). *P*<0.05 was considered statistically significant and were not corrected for multiplicity. The relationship between FMD, pupillometry, SNFD, platelet aggregation, and PSG variables were tested using Pearson‐*r* correlations.

## Results

Thirteen (6 females, 7 males) arteries of the 15 tonsil sets demonstrated immunoreactivity to tyrosine hydroxylase antiserum and were of comparative size. Although the remaining 2 arteries showed immunoreactivity to tyrosine hydroxylase, they were much smaller and were not used for comparison as 3 consistent ROIs could not be determined. Mean (SD) anthropometric, FMD, pupillometry, and platelet aggregation values are reported in Table 1 and mean (SD) PSG values in Table 2. The correlation between the latter variables with SNFD are also included in Tables 1 and 2. For completeness, all the platelet aggregation variables are reported in Table 1. However, because the platelet aggregation agonists were highly intercorrelated, only the aggregation variable has been reported in subsequent tables.

**Table 1:** Mean (SD) Anthropometric, FMD, Pupillary Light Reflex, Platelet Aggregation, and SNFD Values Together With SNFD Pearson‐*r* Correlation Values (N=13)

Variable	Mean (SD)	SNFD Correlation Values
Anthropometric		
Age, y	11.1 (4.1)	0.24
Height, cm	148.2 (22.5)	0.25
Weight, kg	54.4 (30.7)	0.22
BMI kg/m̂2	22.6 (6.8)	0.24
BMI (%ile)	76.7 (31.6)	0.11
Flow‐mediated dilatation		
Resting brachial artery diameter, cm	0.29 (0.06)	0.22
Resting peak systolic velocity, mm/s	113.4 (34.1)	0.64^a^
Resting velocity time integral, mm^2^	20.4 (12.9)	0.63^a^
FMD_60s_, %	5.50 (5.90)	−0.04
FMD_Max_, %	9.22 (5.90)	−0.09
FMD_Time‐to‐Maximum_, s	68.4 (30.6)	0.41
Heart rate, bpm	72.1 (8.71)	−0.39
Pupillary light reflex		
NPI	4.26 (0.22)	−0.71^b^
Baseline pupillary diameter, mm	6.98 (0.41)	0.33
Constricted diameter, mm	4.41 (0.43)	0.58^a^
Change from baseline diameter, %	0.37 (0.04)	−0.70^a^
Constriction latency, s	0.24 (0.37)	−0.65^a^
Mean constriction velocity, mm/s	3.41 (0.44)	−0.64^a^
Peak constriction velocity, mm/s	5.43 (0.63)	−0.63^a^
Postconstriction dilation velocity, mm/s	1.48 (0.13)	−0.59^a^
Platelet aggregation		
Collagen_AUC_	42.9 (11.0)	0.43
Collagen_Aggregation_	97.1 (16.8)	0.47
Collagen_Velocity_	14.3 (3.4)	0.67^a^
lADP_AUC_	19.2 (11.1)	0.50
lADP_Aggregation_	35.8 (18.3)	0.52
lADP_Velocity_	5.23 (1.92)	0.36
TRAP_AUC_	72.3 (14.9)	0.34
TRAP_Aggregation_	109.7 (21.1)	0.27
TRAP_Velocity_	19.8 (4.8)	0.50
ASP_AUC_	46.4 (17.4)	0.46
ASP_Aggregation_	74.9 (24.9)	0.34
ASP_Velocity_	13.4 (5.2)	0.57
ADP_AUC_	43.4 (12.0)	0.52
ADP_Aggregation_	71.8 (18.4)	0.43
ADP_Velocity_	11.7 (3.17)	0.65^a^
SNFD (pixel per region of interest)	2278 (370)	

ADP indicates adenosine diphosphate; ASP, aspirin dialuminate; AUC, area under the curve; BMI, body mass index; FMD, flow‐mediated dilatation; lADP, low‐dose adenosine diphosphate; NPI, Neuronal Pupillary Index; SNFD, sympathetic nerve fiber density; TRAP, thrombin receptor activating peptide.

a *P*<0.05.

b *P*<0.01.

**Table 2:** Mean (SD) PSG Values and Correlation With SNFD (N=13)

PSG Variable	Mean (SD)	Pearson‐*r* Correlation Values With SNFD
Total sleep time, min	418.8 (55.7)	−0.19
NREM 1 (%TST)	12.6 (7.7)	0.19
NREM 2 (%TST)	38.7 (10.4)	−0.03
SWS (%TST)	33.1 (13.2)	−0.36
REM (%TST)	15.6 (4.6)	0.05
Arousals/h sleep time^a^	21.2 (18.3)	0.05
Spontaneous arousals/h sleep time^a^	10.6 (4.5)	0.22
Respiratory arousal/h sleep time^a^	10.3 (18.8)	−0.02
Central apnea hypopnea index^a^	0.45 (0.43)	−0.03
Obstructive apnea hypopnea index^a^	11.8 (24.0)	−0.08
Average SpO_2_desat (%TST)	96.2 (1.2)	−0.17
Average SpO_2_desat (%REM)	96.7 (0.9)	−0.05
Average SpO_2_desat (%NREM)	96.2 (1.1)	−0.13
Nadir SpO_2_, %	89.7 (4.3)	−0.07
REM Nadir SpO_2_, %	92.4 (2.5)	−0.25
NREM Nadir SpO_2_, %	90.7 (4.7)	−0.07

a Spearman‐rho correlation values. NREM indicates non‐rapid‐eye movement; PSG, polysomnography; REM, rapid‐eye movement; SNFD, sympathetic nerve fiber density; SpO_2_, arterial oxygen saturation; SWS, slow wave sleep; TST, total sleep time.

### Correlational Analyses

#### Sympathetic nerve fiber density

A higher SNFD was significantly correlated with a higher resting VTi and resting peak systolic velocity PSV; a lower Neuronal Pupillary Index and a smaller pupillary percent change from baseline diameter, and slower mean pupillary constriction latency, mean pupillary constriction velocity, peak pupillary constriction velocity, and postpupillary constriction dilation velocity; and higher Collagen_Velocity_ and ADP_Velocity_ (Table 1). No significant correlation was observed between any PSG variable and SNFD (Table 2).

#### FMD and pupillometry

The correlation between FMD and pupillometry variables are reported in Table 3. Magnitudes of the significant correlations were all high (*r*>0.59). A larger baseline pupillary diameter was associated with a smaller resting brachial artery diameter whereas a faster postconstriction pupillary dilation velocity was associated with a longer FMD_Time‐to‐Max_, lower resting VTi and lower resting PSV. A greater percent change from pupillary baseline diameter, mean pupillary constriction velocity, and peak pupillary constriction velocity were all associated with a lower resting VTi. A larger constricted diameter and faster peak pupillary constriction velocity were both associated with a lower resting PSV.

**Table 3:** Correlations Results Between Pupillary Light Reflex and FMD Variables

Pupillometry (N=12)	FMD					
	Resting Brachial Artery Diameter	Resting Velocity Time Integral	Resting Peak Systolic Velocity	FMD_60s_	FMD_Max_	FMD_Time‐to‐Maximum_
NPI	−0.27	−0.40	−0.31	0.15	0.12	−0.13
Baseline pupillary diameter	−0.76^b^	0.37	0.50	−0.39	−0.47	0.21
Constricted diameter	−0.59	0.48	0.59	−0.43	−0.50	0.24
Percent change from baseline diameter	0.26	−0.62^a^	−0.55	0.36	0.38	−0.23
Constriction latency	−0.07	0.19	−0.17	−0.23	−0.22	0.04
Mean constriction velocity	0.19	−0.77^b^	−0.51	0.32	0.24	0.11
Peak constriction velocity	0.25	−0.67^a^	−0.71^b^	0.28	0.14	−0.40
Postconstriction dilation velocity	−0.01	−0.80^b^	−0.78^b^	−0.23	−0.33	−0.66^a^

FMD indicates flow‐mediated dilatation; NPI, Neuronal Pupillary Index.

a *P*<0.05.

b *P*<0.01.

Inspection of the association between the FMD variables also revealed a strong association between brachial artery blood flow velocity parameters, i.e. VTi and PSV, and a marker of vascular compliance, i.e. FMD_Time‐to‐Max_ (VTi versus FMD_Time‐to‐Max_, *r*=0.70; *P*<0.01; PSV versus FMD_Time‐to‐Max_, *r*=0.72; *P*<0.01).

#### Platelet aggregation and pupillometry

The correlation between platelet aggregation and pupillometry variables are reported in Table 4. In general, greater platelet aggregation was associated with a longer pupillary constriction latency and a slower mean and peak constriction velocity. The latter significant correlations were all large in magnitude (*r*>0.60).

**Table 4:** Correlations Results Between Platelet Aggregation, Pupillary Light Reflex, and FMD Variables

Platelet Aggregation	Pupillometry (N=12)								FMD (N=13)					
	NPI	Baseline Pupillary Diameter	Constricted Diameter	Percent Change From Baseline Diameter	Constriction Latency	Mean Constriction Velocity	Peak Constriction cVelocity	Postconstriction Dilation Velocity	Resting Brachial Artery Diameter	Resting Velocity Time Integral	Resting Peak Systolic Velocity	FMD_60s_	FMD_Max_	FMD_Time‐to‐Maximum_
Collagen_Aggregation_	−0.60	−0.17	0.13	−0.41	0.65^a^	−0.60^a^	−0.77^b^	−0.38	0.30	0.64^a^	0.43	−0.03	0.18	0.47
lADP_Aggregation_	−0.57	−0.00	0.20	−0.37	0.63^a^	−0.39	−0.52	−0.34	0.17	0.36	0.35	0.14	0.19	0.25
TRAP_Aggregation_	−0.47	−0.42	−0.14	−0.17	0.50	−0.35	−0.47	0.11	0.37	0.35	0.17	−0.32	0.1	0.22
ASP_Aggregation_	−0.67^a^	0.18	0.43	−0.60^a^	0.69^a^	−0.61^a^	−0.75^a^	−0.60^a^	0.20	0.23	0.09	−0.12	−0.10	0.23
ADP_Aggregation_	−0.65^a^	−0.31	−0.14	−0.28	0.79^b^	−0.54	−0.65^a^	−0.13	0.21	0.35	0.27	−0.14	0.05	0.31

ADP indicates adenosine diphosphate; ASP, aspirin dialuminate; FMD, flow‐mediated dilatation; lADP, low‐dose adenosine diphosphate; NPI, Neuronal Pupillary Index; TRAP, thrombin receptor activating peptide.

a *P*<0.05.

b *P*<0.01.

#### Platelet aggregation and FMD

The correlation between platelet aggregation and FMD variables are reported in Table 4. The correlations tended to be small in magnitude, and only a single significant correlation was observed with greater collagen aggregation associated with a higher resting VTi.

## Discussion

This study evaluated the relationship between functional sympathetic activity and structural markers of sympathetic innervation with functional vascular parameters in children with SDB. We found that increased SNFD of the dorsal lingual artery of the tonsil was associated with functional markers of increased sympathetic tone as measured by pupillometry and reduced vascular compliance, as indicated by higher resting blood flow velocity and a longer time to maximal dilatation of the brachial artery. In addition, we found that children with increased sympathetic activity had evidence of increased endothelial damage as measured using platelet aggregation. In summary, these findings suggest that increased sympathetic activity in children with SDB is associated with both structural and functional vascular change.

The strong association that was observed between resting VTi/PSV and the time to maximal brachial artery dilatation suggests that vascular compliance dynamics are altered in children with SDB, which is consistent with previous findings by our group.^9^ Our group has previously reported increased brachial artery blood flow velocity, both during rest and hyperemia, in nonobese children with mild SDB compared with healthy nonsnoring children.^9^ In a separate cohort, we have also observed that children with SDB compared with controls had increased PSV in the ascending aorta using cardiac magnetic resonance imaging.^45^ The strong positive correlation observed between SNFD and FMD measures suggests that the vascular changes present in children with SDB are systemic. Furthermore, the enhanced innervation of the blood vessels may lead to an increase in the release of the vasoconstrictor neurotransmitter, noradrenaline, and consequently reduce compliance of the vessels.^22^ This would explain our previous report of increased blood flow velocity in children with SDB and concurs with the increase in velocity also demonstrated in the middle cerebral artery by others.^29^ More important, the inter‐relationship between increased vascular sympathetic innervation, smooth muscle cell hyperplasia,^22^ and extracellular matrix protein proliferation, which promotes vascular remodeling, may explain our previous report of delayed dilation response in the brachial artery of young children with mild SDB and is corroborated by the delayed dilation response to hyperemia in metacarpal microvasculature in children with SDB demonstrated by a different group.^46^ A strength of the FMD approach utilized by the present study is that continuous measurements were collected over a 3‐minute time frame allowing for a complete evaluation of the dilatation response and hence why we were able to demonstrate such a robust relationship with other variables. This is in contrast to the standard approach, which is to limit measurement to 60 seconds.

The pupillary light reflex is considered a robust measure of autonomic nervous system function.^32^, ^36^, ^38^ In this study, we found that greater SNFD was associated with higher sympathetic tone as measured by pupillometry (eg, decreased mean and peak pupillary constriction velocity and a reduced percentage change in pupil diameter in response to a light stimulus). Reduced peak constriction velocity and delayed time to minimum constriction diameter are considered evidence of abnormal pupillary and hence autonomic nervous system function.^32^, ^38^ These findings complement similar associations we observed between SNFD and FMD parameters.

Increased platelet aggregation is considered a marker of vascular dysfunction.^18^ Our results show that the children with increased VTi also exhibit increased platelet aggregation in response to the collagen antigen. Collagen‐sensitive aggregation suggests that platelets have come into contact with collagen.^47^ In healthy subjects, collagen in the vessel wall is concealed by the endothelial cells that line the lumen, but injury to the vessel wall (possibly from increased shear stress) may facilitate platelet/collagen contact.^48^ In addition, our finding of a strong association between the pupillary light reflex variables and platelet aggregation, in particular to the collagen antigen, further supports the close relationship between augmentation of sympathetic tone, reduced vascular compliance, and functional platelet changes suggestive of intimal injury.

Increased SNFD has been reported in early development in spontaneously hypertensive rats^22^ and in rats exposed to hypoxia in utero and who subsequently developed early‐onset arterial hypertension as they matured.^24^ In mice, sleep fragmentation has been reported to increase peak blood flow and delay the dilatation response in the dorsal tail vein and promotes elastic fiber disorganization in both the aortic arch and thoracic aorta.^21^ Whether the evidence presented in this study supports the current paradigm, that a persistent increase in sympathetic activity in early childhood predisposes adults to early hypertension requires further investigation. The use of pupillometry and FMD, as reported in this article, may play a role in the early detection of vascular changes in children with SDB. This could potentially help identify children with SDB at lesser or greater risk of vascular change, thereby facilitating early intervention and those who require ongoing review.

The range of brachial VTi results both at rest and during hyperemia in our previous study varied from normal to high, suggesting that not all children with SDB exhibited the increased VTi phenotype.^9^ This heterogeneity in vascular response has been noted in studies looking at the effects of aging, disease, and therapeutic interventions on vascular response.^49^ Whether the chronicity of SDB or its severity are the more‐important contributors to increased vascular sympathetic innervation remains to be determined. It also raises the possibility of underlying genetic factors or lifestyle factors that may protect or impede vascular integrity in some children with SDB.

### Limitations

This study could have been further strengthened by larger subject numbers, which would also allow for stratification by narrower age bands.^41^ However, given the practical challenges of collecting multiple measures in young children, this is a comprehensive evaluation of vascular function and structure. Further studies will be required to optimize these findings so that these techniques may be used as part of a clinical assessment. A potential criticism of the study is that the findings may be explained by local inflammatory changes within the tonsillar tissue. However, this is unlikely to explain the increased SNFD observed in the dorsal lingual artery and the pattern correlations observed between SNFD and both pupillometry and FMD. Our study found no relationship between conventional PSG measures of SDB severity and vascular compliance/sympathetic function and structure. It is possible that conventional PSG measures recorded on a single night may not be the appropriate parameters to reflect the full extent of the severity of SDB occurring night after night on vascular development or that SDB has a heterogeneous impact on vascular development, which may arise from genetic variability.^50^

A persistent increase in sympathetic tone is an integral factor in hypertension both in children^10^ and adults.^51^ Our results are clinically significant considering that most children with SDB remain underdiagnosed and untreated; we postulate that this may well be an unrecognized risk factor for hypertension in adult life and implies that SDB may have a more‐deleterious effect on children's vascular health than previously recognized.

In conclusion, this is the first study to demonstrate that arterial structural changes are correlated with functional vascular changes and that both are associated with sympathetic tone. The long‐term implications of increased resting blood flow velocity and potentially the resultant increase in shear stress on a child's developing vasculature are currently unknown, but the changes we have demonstrated may be a harbinger of accelerated cardiovascular disease in later years.

## Author Contributions

Study design: all authors. Data collection: Kontos, Wabnitz. Data Analysis: Kontos, Green. Interpretation of results, Kontos, Lushington, Kennedy. Preparation of manuscript: all authors.

## Sources of Funding

Financial support for this study was provided by the Channel 7 Research Foundation and the Women's and Children's Hospital Foundation.

## Disclosures

None.
